# Cannabinoids in hematologic malignancies: mechanisms and therapeutic potential for overcoming drug resistance

**DOI:** 10.3389/fphar.2026.1921501

**Published:** 2026-09-25

**Authors:** Sixu Li, Xiaokun Jian, Yufu Tang, Zhentao Wang, Tianjiao Min, Luyao Yu, Beier Jiang, Ying He

**Affiliations:** 1 College of Food Science and Technology, Shanghai Ocean University, Shanghai, China; 2 Navy Medical Center, Naval Medical University, Shanghai, China

**Keywords:** ABC transporters, cannabinoids, combination therapy, drug resistance, hematologic malignancies

## Abstract

Hematologic malignancies are highly heterogeneous diseases in which the emergence of therapy resistance remains a major obstacle to durable clinical benefit. Cannabinoids, acting through the endocannabinoid system, have drawn increasing interest because of their potential to modulate multiple resistance-associated pathways, yet the available evidence remains fragmented across disease entities, experimental models, and mechanistic frameworks. In this narrative review, we examine the molecular mechanisms by which cannabinoids may counteract drug resistance in leukemia, lymphoma, and multiple myeloma. We discuss their effects on ABC transporter-mediated drug efflux, mitochondrial apoptosis, redox homeostasis, endoplasmic reticulum stress and autophagy, pro-survival signaling, and tumor microenvironment-mediated protection. We also evaluate preclinical evidence for cannabinoid-based combinations with conventional chemotherapy, targeted therapies, immunotherapies, and radiotherapy. Although *in vitro* and animal studies indicate that cannabinoids may reverse resistant phenotypes and enhance the activity of existing treatments, the translational relevance of these findings is limited by the predominance of cell-line and xenograft models, the paucity of clinical evidence for antitumor efficacy, pharmacokinetic constraints, potential drug–drug interactions, and context-dependent effects that may be neutral or adverse in certain immunological settings. Thus, while the endocannabinoid system represents a potential therapeutic target for sensitizing resistant hematologic malignancies, clinical translation will require validation at pharmacologically achievable exposures, the use of immunocompetent and clinically relevant models, identification of predictive biomarkers, and rigorous evaluation in prospective clinical studies.

## 1 Introduction

Hematologic malignancies represent a highly heterogeneous group of malignant diseases originating from hematopoietic stem/progenitor cells or the lymphatic system (Auberger et al., 2020). They are characterized by rapid proliferation, strong invasive potential, and persistent clonal evolution, features that underlie their marked biological complexity (Capelletti et al., 2024). Even if treatment is effective at the beginning, subsequent treatments can still alter the cellular structure (Landau et al., 2014). Even when remission is achieved after the initial therapy, subsequent treatment can continue to shape clonal architecture by selecting resistant populations and leading to an accumulation of genetic and epigenetic changes (Hata et al., 2016). Over time, these changes may lead to the emergence of resistant sub-clones, leading to the recurrence and undermining long-term treatment effects (Jan and Majeti, 2013). Thus, drug resistance is now widely recognized as one of the main reasons why malignant tumors of the blood system are difficult to treat and prone to recurrence.

In recent years, there have been many breakthroughs in the treatment of blood cancers, and more treatment options are now available. However, the main treatment methods still have several drawbacks: the effects do not last long, and the cells are prone to developing resistance, leading to disease recurrence (Zielonka and Jamroziak, 2024). Traditional chemotherapy is not precise enough and can inadvertently damage healthy cells, including normal hematopoietic and immune cells (Spencer Chapman et al., 2024). As a result, patients often experience side effects such as bone marrow suppression, infections, and gastrointestinal discomfort, which make it difficult for them to continue treatment, and the treatment often becomes intermittent (Hurkmans et al., 2022). Stem-cell transplantation could potentially cure the disease, but donors are hard to find, the transplant risk is high, and there is also a possibility of graft-versus-host disease (Shane, 2024). It is, therefore, not widely used. Chimeric antigen receptor T-cell (CAR-T) therapy has indeed made significant progress in treating relapsed and refractory leukemia and lymphoma. However, it is too expensive, complicated to produce, and has significant side effects. It may also lead to recurrence due to antigen escape (Kostelecka et al., 2024). Therefore, developing innovative therapeutic strategies that can effectively mitigate or overcome therapy resistance remains an urgent task in the field of hematologic malignancy treatment.

Cannabinoids exert their biological effects through the endocannabinoid system (ECS), particularly via cannabinoid receptors (CBRs) (Gado et al., 2021). In oncology, the clinical application of cannabinoids has primarily focused on alleviating side effects associated with cancer treatment, such as chemotherapy-induced nausea and vomiting, moderate to severe cancer pain, and cachexia. Some studies have demonstrated their apparent efficacy in improving the patients’ quality of life (Aguzzi et al., 2024). With a deeper understanding of the ECS, research indicates that cannabinoids not only exert adjuvant therapeutic effects but also directly modulate tumor-related signaling pathways and stress-response programs relevant to resistance. The high expression of cannabinoid receptor 2 (CB2) receptors in hematopoietic and immune compartments lays a crucial foundation for cannabinoids to exert relatively selective biological effects in hematologic malignancies (Raitio et al., 2005). Preclinical studies indicate that cannabinoids may modulate therapy resistance via multi-target mechanisms. These multi-pathway mechanisms involve key biological processes, including apoptosis and autophagy regulation, cell-cycle arrest, drug transport, and interactions with the tumor microenvironment (Hinz and Ramer, 2022). For instance, cannabidiol (CBD) has been shown *in vitro* to downregulate *ABCB1* gene expression and inhibit P-glycoprotein (P-gp)-mediated drug efflux, an effect that was associated with enhanced bortezomib sensitivity in resistant multiple-myeloma cells (Iffland and Grotenhermen, 2017). Beyond their direct effects on tumor cells, the endogenous cannabinoid 2-arachidonoylglycerol (2-AG) has been reported to indirectly modulate resistance phenotypes in preclinical models by altering immune and stromal cell functions in the tumor microenvironment (Yan et al., 2023). These observations indicate that cannabinoids may influence therapy resistance in hematologic malignancies at several levels and may, therefore, hold value as components of combination treatment strategies. Collectively, the ECS is emerging as a potentially actionable system for improving treatment sensitivity and extending the durability of existing therapies. Recently, there has been increasing interest in using cannabinoids to address the issue of resistance to blood-cancer treatments. However, the existing research data are scattered across various areas—some focusing on different diseases, others using different experimental models, and still others offering explanations from different perspectives. This makes it difficult to gain a comprehensive understanding. Therefore, this review aims to discuss the molecular pathways underlying resistance, examine whether cannabinoids can be used in combination with traditional antineoplastic drugs, and summarize current research findings on leukemia, lymphoma, and multiple myeloma. By combining these mechanisms with preclinical studies, we hope to understand how the ECS signaling pathway and resistance interact. At the same time, we will identify areas that require special attention when developing cannabinoid-based combined treatment regimens for blood cancer.

## 2 The molecular mechanisms by which cannabinoids overcome treatment resistance in hematological malignancies

### 2.1 Cannabinoids restore intracellular drug accumulation by modulating ABC transporter-mediated drug efflux

The ATP-binding cassette (ABC) transporters are key mediators of drug resistance in blood cancers (Salvia et al., 2017). These proteins, powered by adenosine triphosphate (ATP), can actively efflux chemotherapy drugs from cells, reducing the amount of medication inside the cell to a level where it is no longer effective at killing cancer cells (Hassanpour et al., 2017). As a result, treatment fails, leading to disease relapse. Among these proteins, P-glycoprotein (P-gp), multidrug resistance-associated protein 1 (MRP1), and breast cancer resistance protein (BCRP) have been studied the most. They are particularly important in leukemia and lymphoma, where these cancers are less sensitive to chemotherapy, leading to poor treatment outcomes (Stefan et al., 2017). There is increasing evidence that cannabinoids may contribute to regulate the activity or expression of these proteins, thereby inhibiting drug efflux.

#### 2.1.1 P-gp

The *ABCB1* gene (also known as *MDR1*) encodes P-gp, a member of the ABC transporter family. It is an ATP-dependent efflux transporter that transports various chemotherapy drugs out of the cell. These drugs include common medications such as vincristine, doxorubicin, and daunorubicin (Kimchi-Sarfaty et al., 2007). P-gp reduces the intracellular concentration of these drugs to non-toxic levels, leading to multi-drug resistance in blood cancers. Several *in vitro* studies indicate that certain phytocannabinoids and synthetic cannabinoids may render resistant cancer cells sensitive to chemotherapy by regulating the expression and function of P-gp. The majority of these data are derived from studies utilizing Δ-9-Tetrahydrocannabinol (THC) (a CB1/CB2 partial agonist), CBD (a non-psychoactive phytocannabinoid with low CB1/CB2 affinity), and WIN-55,212-2 (a synthetic full CB1/CB2 agonist)—three compounds that differ substantially in receptor selectivity, intrinsic efficacy, and lipophilicity, and they may, therefore, engage P-gp through overlapping but non-identical mechanisms. In vincristine-resistant leukemia CEM/VLB100 cells, prolonged exposure to THC or CBD reduced P-gp protein expression, increased intracellular Rhodamine 123 accumulation, and enhanced sensitivity to vincristine (Malhotra et al., 2021). Furthermore, studies have shown that after 72 h of treatment, cannabinol, CBD, and THC all reduced P-gp expression in CEM/VLB100 cells by up to 50%; WIN-55,212-2 had a similar effect (Holland et al., 2006).

Beyond transcriptional regulation, certain cannabinoids—particularly the highly lipophilic phytocannabinoids THC and CBD—can directly interfere with P-gp function. These compounds are highly lipophilic and can readily insert into lipid bilayers. This alters membrane fluidity and modulates the lipid environment, thereby impacting the conformation and function of transport proteins (Oz et al., 2022). This changes the fluidity of the cell membrane, which, in turn, affects the surrounding fatty environment. As a result, both the shape and function of transport proteins are impacted. Supporting this possibility, molecular docking, molecular dynamics simulations, and functional assays have shown that CBD interacts with the transmembrane drug-binding region of P-gp and inhibits substrate efflux (Mora Lagares et al., 2020). In this way, the substrate is trapped within the cell and cannot be released. Moreover, phytocannabinoids may also disrupt other less common drug excretion pathways. Studies on T-cell acute lymphoblastic leukemia (T-ALL) cells have found that CBD disrupts the function of extracellular vesicles, the “delivery carriers,” preventing the drugs from being excreted (Shie et al., 2025). Nevertheless, cannabinoid-mediated regulation of P-gp does not appear to be uniformly suppressive: short-term exposure to CBD or THC has also been associated with transient upregulation of ABCB1 mRNA (Arnold et al., 2012). Taken together, these observations indicate that the effects of cannabinoids on P-gp are dynamic and strongly dependent on exposure conditions and cellular context.

#### 2.1.2 MRP1

The MRP1 protein belongs to the ABCC family and plays a key role in multi-drug resistance (Dean, 2002). It helps expel several antineoplastic drugs from cells, particularly those used to treat blood cancers, such as vincristine, anthracyclines, and mitoxantrone (Čižmáriková et al., 2025). Interestingly, MRP1 can also transport drug complexes out of cells in the presence of reduced glutathione (Yang et al., 2024). This enhances the cells’ ability to detoxify and improves the effectiveness of drug elimination. In cells of acute myeloid leukemia (AML), non-Hodgkin lymphoma (NHL), and T-ALL, MRP1 expression increases, often indicating poor chemotherapy outcomes and a poor prognosis (Ebner et al., 2023). Current evidence indicates that phytocannabinoids, particularly CBD, can reverse MRP1-mediated drug resistance primarily through direct inhibition of transporter activity (Holland et al., 2008). Experiments have shown that in cells with excessive MRP1, CBD, THC, and cannabigerol (CBG) can inhibit drug efflux at varying concentrations, with CBD being the most effective (Schmitt et al., 2016).

Further research indicates that CBD and MRP1 can indeed form a direct connection. CBD and MRP1 interact functionally, and MRP1 contains a hydrophobic pocket (H-pocket) that serves as a substrate-binding site (Conseil et al., 2019). This allows it to stabilize interactions that could hinder conformational changes during substrate transport. This substance can also disrupt ATP hydrolysis, preventing chemotherapy drugs from being excreted. Not only is the function of transport proteins inhibited by CBD, but other pathways for drug excretion may also be blocked. Experiments further indicated that MRP1 can sequester chemotherapeutic agents within intracellular vesicles (Holland et al., 2008). Therefore, it appears that cannabinoids can block both pathways for drug excretion, making them quite effective in addressing resistance issues related to MRP1.

#### 2.1.3 BCRP

BCRP is simply a dimeric half-carrier within the ABCG family of molecules (Taylor et al., 2017). It plays a crucial role in the fight against blood cancers. This substance can transport a variety of antineoplastic drugs, including mitoxantrone and topotecan (Mseddi et al., 2026). Clinical studies have found that patients with AML and acute lymphoblastic leukemia (ALL) often exhibit excessive expression of BCRP (Abbott et al., 2002). Clinical studies have shown that high BCRP expression in leukemia patients is consistently associated with poorer outcomes, particularly with respect to disease relapse and shorter disease-free survival. Available evidence further indicates that phytocannabinoids may directly inhibit BCRP function. Experiments show that in BCRP-overactive cells, components such as THC, CBD, and cannabinol (CBN) cause an accumulation of mitoxantrone within the cells, and the effect is proportional to their concentrations (Holland et al., 2007).

Mechanistic analyses demonstrated that these phytocannabinoids inhibited both basal and substrate-stimulated ATPase activity, indicating that they suppress the energy-dependent transport cycle of BCRP. At sub-toxic concentrations, THC, CBD, and CBN also restored sensitivity of BCRP-overexpressing cells to mitoxantrone and topotecan, producing resistance-reversing effects comparable to those of established BCRP inhibitors. Notably, prolonged cannabinoid exposure did not alter BCRP protein expression, indicating that the observed chemosensitization primarily results from functional inhibition rather than reduced transporter abundance (Holland et al., 2007). Because BCRP is also highly expressed in cancer stem-cell populations and is closely linked to stemness and drug resistance, its inhibition by cannabinoids may have broader implications for limiting refractory disease and relapse.

Overall, current evidence indicates that cannabinoids are thought to modulate ABC transporter-mediated drug resistance through multiple mechanisms, including regulation of transporter expression, inhibition of ATPase-dependent transport activity, interference with substrate efflux, and suppression of vesicle-mediated drug export. However, the current evidence remains predominantly derived from *in vitro* models, and the magnitude and direction of these effects may vary with cannabinoid concentration, exposure duration, and cellular context. Further *in vivo* and clinical studies are therefore needed to define therapeutically relevant dosing, pharmacokinetic behavior, safety, and optimal combinatorial strategies with conventional antineoplastic therapies. A more detailed structural understanding of transporter–cannabinoid interactions may also facilitate the development of selective cannabinoid-based modulators for overcoming multi-drug resistance in hematologic malignancies. A schematic diagram summarizing the relevant mechanisms is shown in Figure 1.

**FIGURE 1:**
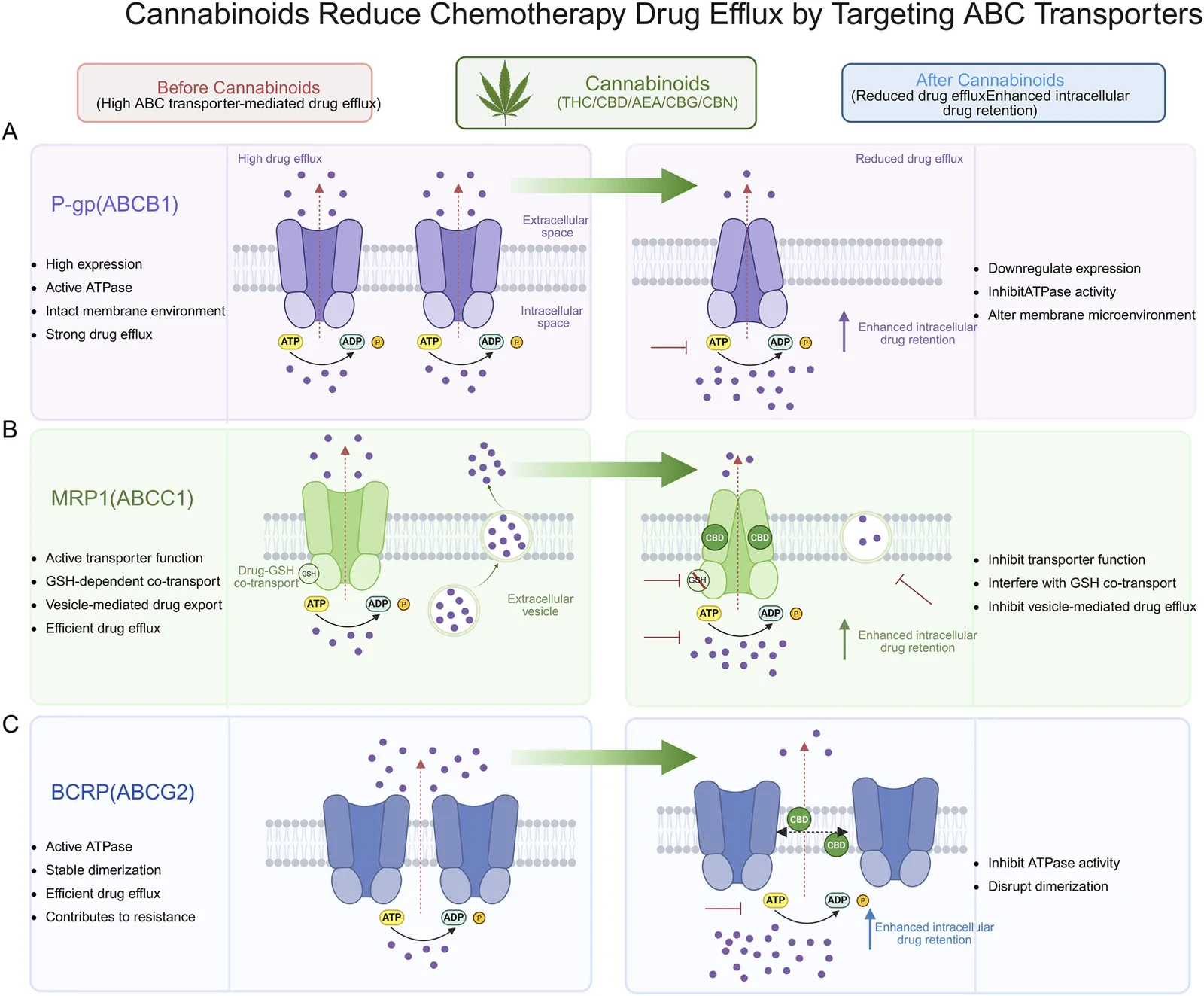
Cannabinoids attenuate ABC transporter-mediated chemotherapy efflux. The left panels depict the pre-treatment state (high transporter activity), while the right panels illustrate the post-treatment state following cannabinoid exposure (reduced drug efflux and enhanced intracellular drug retention). Small filled circles represent chemotherapeutic drug molecules, wavy arrows indicate directional drug transport across the plasma membrane, and the ATP/ADP symbols denote the energy source hydrolyzed by each transporter to power active efflux. **(A)** P-glycoprotein (P-gp). Under baseline conditions, P-gp is highly expressed at the plasma membrane, exhibits active ATPase activity, and maintains an intact lipid microenvironment, collectively driving robust drug efflux. Cannabinoids counteract this by (i) downregulating P-gp protein expression, (ii) directly inhibiting its ATPase activity, and (iii) altering the plasma membrane lipid microenvironment, which destabilizes transporter conformation and function. **(B)** Multidrug resistance-associated protein 1 (MRP1). MRP1 mediates drug resistance through three complementary mechanisms: ATP-dependent direct drug export, glutathione (GSH)-dependent co-transport, and vesicle-associated drug sequestration and elimination. Cannabinoids—particularly CBD—inhibit MRP1 function by interfering with the drug–GSH co-transport coupling and suppressing vesicle-mediated drug export. **(C)** Breast cancer resistance protein (BCRP). BCRP functions as a homodimer and relies on ATPase activity for drug extrusion. Cannabinoids impair BCRP-mediated efflux by (i) inhibiting ATP hydrolysis and (ii) disrupting the stable dimer interface required for functional transport. Collectively, these multi-level inhibitory mechanisms converge to enhance intracellular drug retention and restore chemosensitivity in drug-resistant hematologic malignancies.

### 2.2 Cannabinoids re-prime mitochondrial apoptosis and override apoptotic evasion

Apoptotic evasion is a central mechanism of therapeutic resistance in hematologic malignancies. In AML, B-cell lymphoma, and multiple myeloma, the overexpression of anti-apoptotic BCL-2 family proteins, including BCL-2, BCL-XL, and MCL-1, increases the mitochondrial threshold for apoptotic commitment, thereby enabling malignant cells to survive chemotherapy- or targeted therapy-induced stress (Touzeau et al., 2016). A hallmark of acquired resistance is the reduction of mitochondrial apoptotic priming, whereby resistant cells become less responsive to intrinsic apoptotic stimuli and require stronger death signals to undergo irreversible cell death (Bhatt et al., 2020).

Evidence from multiple preclinical studies shows that phytocannabinoids can reinitiate apoptosis in resistant tumor cells. In leukemia studies, CBD can activate intrinsic apoptotic pathways in Jurkat and Molt-4 cells (McKallip et al., 2006). This leads to the loss of mitochondrial membrane potential, followed by activation of a cascade. More notably, the response of Jurkat ALL cells to CBD is unique: at low concentrations, it causes slight proliferation; at high concentrations, it targets the mitochondria and initiates a caspase-9/caspase-3 cascade (Olivas-Aguirre et al., 2019). These findings indicate that phytocannabinoids may directly interfere with the mitochondrial death signaling pathway, bypassing the mechanisms that tumor cells use to resist apoptosis.

Later studies have also found that metabolic issues may play a key role in this process. CBD can reduce cell survival in THP-1 mononuclear leukemia cells and impair mitochondrial respiratory capacity (Pereira et al., 2021). This indicates problems with oxidative phosphorylation in the cells, leading to an imbalance in energy levels. This effect may undermine the metabolic support necessary to maintain cell apoptosis resistance and thereby reactivate mitochondrial function. These findings indicate that mitochondrial permeability transition is likely one of the mechanisms by which phytocannabinoids may reactivate mitochondrial apoptosis.

Cannabinoid-induced mitochondrial apoptosis has also been documented in lymphoma and myeloma models. In mantle cell lymphoma, cannabinoid receptor activation promoted ceramide accumulation, p38 mitogen-activated protein kinase (MAPK) phosphorylation, mitochondrial membrane depolarization, and caspase activation, consistent with engagement of a ceramide–p38 MAPK–mitochondrial death axis (Gustafsson et al., 2006). In multiple myeloma, synthetic cannabinoid derivatives selectively induced apoptosis in myeloma cell lines and patient-derived plasma cells, with minimal toxicity toward normal hematopoietic stem cells (Alghamdi et al., 2025). This response was associated with activation of caspase-2, upregulation of Bcl-2-associated X protein (Bax) and Bcl-2 homologous antagonist/killer (Bak), reduction of anti-apoptotic proteins such as BCL-xL and MCL-1, and modulation of Akt/protein kinase B (PKB) signaling. Partial attenuation of apoptosis by ceramide synthesis inhibition further supports an important role for ceramide-mediated mitochondrial priming in cannabinoid-induced cell death (Herrera et al., 2006).

Overall, current evidence indicates that cannabinoids may lower the apoptotic threshold of drug-resistant hematologic tumor cells by restoring mitochondrial apoptotic priming. However, this mechanism is supported predominantly by *in vitro* and *ex vivo* and evidence and requires further *in vivo* validation. Through direct mitochondrial targeting, modulation of ceramide signaling, disruption of cellular metabolism, and activation of the intrinsic caspase cascade, suppressed death programs may be re-engaged, thereby potentially enhancing the efficacy of conventional chemotherapy and targeted therapies.

### 2.3 Cannabinoids disrupt redox homeostasis and mitochondrial stress adaptation

Drug-resistant hematologic malignancies rely heavily on tightly controlled redox homeostasis and mitochondrial stress adaptation to sustain survival under therapeutic pressure (Lee et al., 2026). Resistant tumor cells typically maintain intracellular reactive oxygen species (ROS) within a permissive range that supports proliferation while avoiding lethal oxidative injury. At the same time, they enhance mitochondrial metabolic capacity and stress-buffering mechanisms, enabling them to tolerate chemotherapy- or targeted therapy-induced damage and prevent progression toward irreversible cellular dysfunction (Prajapati et al., 2025). Accordingly, disruption of redox balance and mitochondrial stress adaptation has emerged as a promising strategy for overcoming therapeutic resistance.

Available preclinical evidence indicates that cannabinoids may interfere with these adaptive programs by perturbing both ROS regulation and mitochondrial function. In leukemia models, CBD activates CB2 receptors, thereby increasing ROS (Massi et al., 2013). This leads to a decrease in mitochondrial membrane potential and the activation of caspases. If drugs block the CB2 signaling, these reactions are significantly reduced, suggesting that cannabinoids play an important role in the process of leukemia cell death (Ceccacci et al., 2024). Overall, oxidative stress is a key factor in the cannabinoid-induced mitochondrial damage in hematological cancer cells. Similar results have been observed in experimental models of lymphoma. After treating B-cell lymphoma cells from dogs and humans with CBD, THC, or synthetic cannabinoid WIN-55,212-2, the survival rate of tumor cells decreased significantly. At the same time, oxidative stress increased, inflammatory signals changed, and mitochondrial function was impaired (Gustafsson et al., 2006). Furthermore, a study has shown that CBD can inhibit the proliferation of human chronic myeloid leukemia K562 cancer cells by inducing apoptosis, reducing mitochondrial membrane potential, and activating caspases 3 and 7 (Anceschi et al., 2022). This further clarifies that oxidative stress is a key reason why cannabinoids cause mitochondrial dysfunction in various types of tumors.

It should be noted that the strength of these effects may be related to the amount of oxygen in the tumor’s surrounding environment. Studies on Jurkat leukemia cells have shown that when the oxygen concentration is similar to that in normal tissues, the cytotoxic effect of CBD is weaker (Kalenderoglou et al., 2017). This indicates that the oxygen content in bone marrow or lymphoid tissue may reduce oxidative stress induced by phytocannabinoids, thereby affecting the cells’ responses to treatment.

Overall, current evidence indicates that cannabinoids may compromise the redox homeostasis and mitochondrial stress tolerance of certain drug-resistant tumor cells under defined experimental conditions. By driving ROS accumulation beyond tolerable thresholds and destabilizing mitochondrial metabolism, cannabinoids shift malignant cells from an adaptive stress state toward irreversible mitochondrial damage and apoptotic commitment. This redox-mediated vulnerability is likely to complement the restoration of mitochondrial apoptotic priming described above, together contributing to re-sensitization of resistant hematologic malignancies to chemotherapy and targeted therapies.

### 2.4 Cannabinoids reprogram ER stress and autophagy networks in drug-resistant hematologic malignancies

Under sustained therapeutic pressure, hematologic malignancies frequently preserve survival by remodeling intracellular stress-response systems that maintain proteostasis. Among these, endoplasmic reticulum stress signaling, the unfolded protein response, and the integrated stress response constitute a central adaptive network that enables tumor cells to tolerate proteotoxic stress imposed by chemotherapy or targeted therapy. By attenuating global protein translation, reallocating metabolic resources, and activating autophagy-dependent quality control, these pathways limit the accumulation of misfolded proteins and buffer cellular damage. In highly secretory malignancies, particularly multiple myeloma and certain lymphoid tumors, endoplasmic reticulum stress signaling is tightly coupled to autophagy and adaptive translational remodeling, thereby providing a major basis for drug persistence (Sedlacek, 2025).

Limited preclinical evidence indicates that cannabinoids may interfere with this adaptive circuitry, shifting the balance between stress responses and autophagy pathways from protecting cells to signaling pathways that lead to cell death. In experimental models of multiple myeloma, exposure to both THC and CBD significantly reduced the growth of U266 and RPMI-8226 cells. Additionally, it increased microtubule-associated protein 1A/1B-light chain 3 (LC3)-II accumulation and decreased p62/sequestosome 1 (p62) levels, indicating enhanced autophagy activity. When autophagy was inhibited with drugs, cell survival was partially restored, indicating that the cytotoxicity caused by phytocannabinoids is at least partially due to a cell death mechanism that relies on autophagy rather than purely protective autophagy (Nabissi et al., 2016). Another interesting finding is that the combination of THC and CBD can enhance the antineoplastic effects of carfilzomib. This suggests that cannabinoids may enhance the ability to kill resistant myeloma cells by disrupting stress-response pathways that maintain protein balance (Ramer and Hinz, 2025).

Related observations have been reported in T-ALL. A phytocannabinoid combination containing CBD, cannabidivarin (CBDV), and the novel compound 331-18A (synthetic cannabinoid derivatives) was shown to perturb intracellular Ca^2+^ homeostasis through CB2 receptors and transient receptor potential vanilloid 1 (TRPV1) channels, leading to the depletion of ER calcium stores and activation of integrated stress-response-related signaling (Besser et al., 2024). These findings indicate that cannabinoid-mediated remodeling of stress networks may originate from upstream Ca^2+^ disequilibrium and ER stress, ultimately promoting broader reprogramming of adaptive survival pathways in resistant hematologic tumor cells.

Although mechanistic studies in hematologic malignancies remain limited, evidence from solid tumors has provided a useful framework for understanding how cannabinoids engage endoplasmic reticulum stress-autophagy signaling. THC has been shown to induce autophagy-mediated tumor-cell death through *de novo* ceramide synthesis, which promotes eIF2α phosphorylation and activation of p8, leading to TRB3 induction and subsequent inhibition of Akt/mTORC1 signaling (Salazar et al., 2009). Subsequent studies further delineated the p8-ATF4–CHOP–TRB3 cascade as a central mechanism linking cannabinoid-induced endoplasmic reticulum stress to autophagy-driven cell death. Importantly, Midkine has emerged as a key modulator of this pathway: elevated Midkine expression suppresses p8/TRB3 induction. It restores Akt/mTORC1 signaling through activation of the ALK axis, thereby attenuating cannabinoid-induced autophagic cytotoxicity (Lorente et al., 2011). Conversely, genetic silencing of Midkine or pharmacologic inhibition of ALK re-sensitizes tumor cells to cannabinoid-induced stress signaling.

Autophagy itself exhibits marked context dependence within tumor stress responses. Under moderate stress, it generally serves a cytoprotective role by facilitating the clearance of damaged organelles and misfolded proteins. However, prolonged or excessive endoplasmic reticulum stress can shift autophagy toward a pro-death program. Sustained activation of endoplasmic reticulum stress pathways has been proposed to drive this transition, and CHOP activation, with subsequent TRB3 induction, can further suppress Akt/mTOR signaling, thereby reinforcing autophagy-associated death pathways (Abrams and Guzman, 2015). In this setting, persistent stress signaling converts autophagy from an adaptive process into a mediator of irreversible cellular injury.

Current research indicates that cannabinoids may regulate the ER stress signaling pathways associated with drug-resistant blood cancers. However, direct evidence in hematologic malignancies remains limited, and several mechanistic insights are derived from solid-tumor models. They have been reported to disrupt calcium homeostasis, activate the stress-response signaling pathway, and alter the balance between cell protection and cytotoxic autophagy. Cannabinoids also turn a highly adaptive protein repair system into an exacerbated stress response, into an induced cell-death in cancer cells. This mechanism might be another way in which cannabinoids can help restore drug efficacy. A schematic diagram summarizing the relevant mechanisms is shown in Figure 2.

**FIGURE 2:**
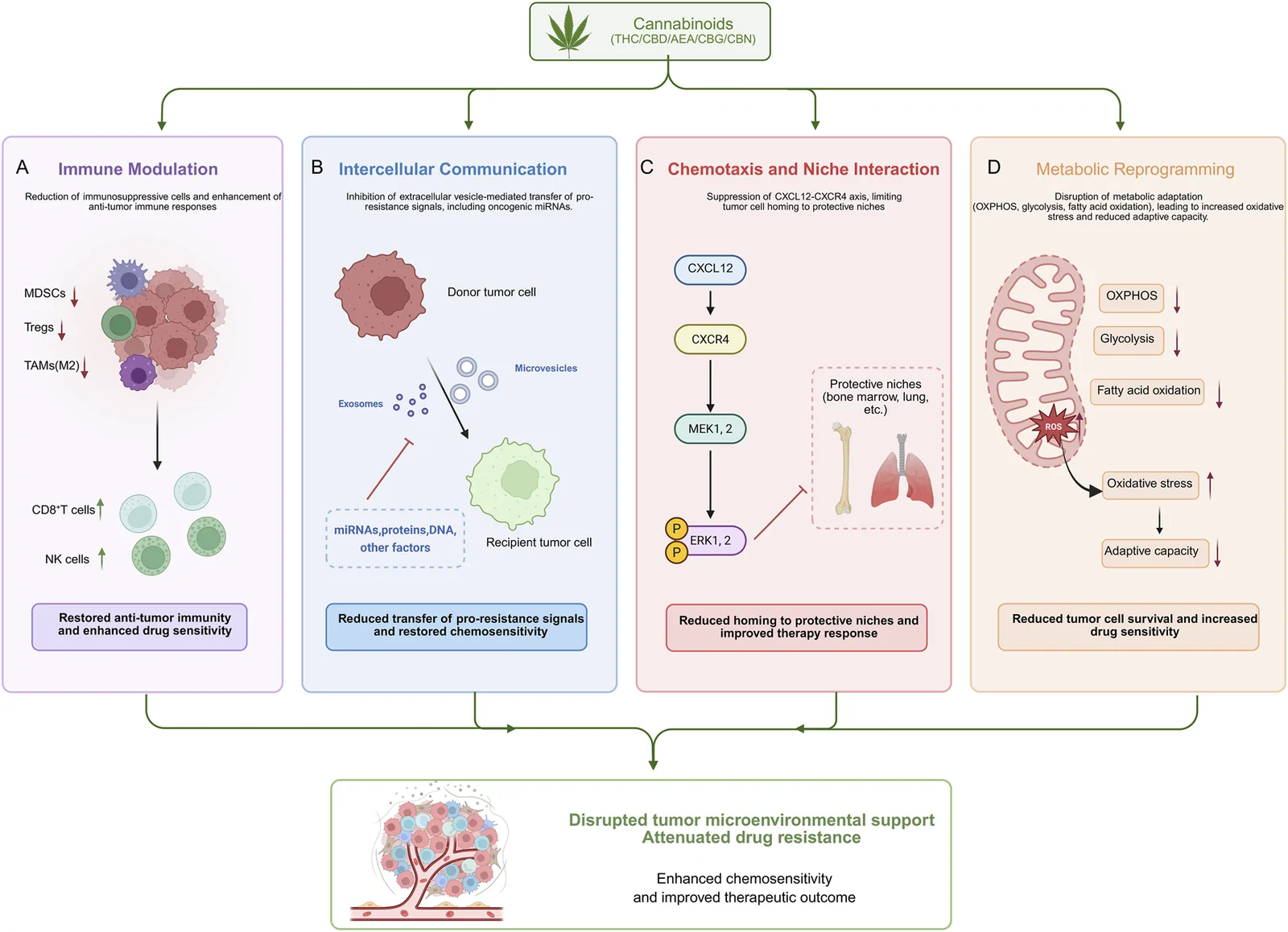
Cannabinoids lower the apoptotic threshold through coordinated regulation of tumor-intrinsic stress and survival pathways. Upward arrows (↑) denote upregulation or activation; downward arrows (↓) denote downregulation or suppression; solid arrows indicate direct activation or conversion. **(A)** Redox disruption. Cannabinoids elevate intracellular reactive oxygen species (ROS) generation while simultaneously impairing the antioxidant defense system—comprising superoxide dismutase (SOD), catalase (CAT), and reduced glutathione (GSH)—thereby triggering oxidative stress. The resulting redox imbalance causes oxidative damage to proteins, lipids, and DNA. **(B)** Mitochondrial apoptosis induction. Cannabinoids trigger the intrinsic apoptotic cascade characterized by loss of mitochondrial membrane potential (ΔΨm), release of cytochrome c from the mitochondrial intermembrane space into the cytosol, and sequential activation of initiator caspase-9 and downstream effector caspases. This process is reinforced by upregulation of pro-apoptotic BCL-2 family members BAX and BAK (↑) and concurrent suppression of the anti-apoptotic protein BCL-2 (↓). **(C)** Endoplasmic reticulum (ER) stress and autophagy reprogramming. Cannabinoids activate the unfolded protein response (UPR) and integrated stress response (ISR) signaling axis, specifically the PERK (protein kinase RNA-like ER kinase) → phosphorylated eIF2α (eukaryotic initiation factor 2 alpha) → ATF4 (activating transcription factor 4) → CHOP (C/EBP homologous protein) cascade. Sustained CHOP upregulation converts initially cytoprotective autophagy into autophagy-dependent cell death. **(D)** Suppression of pro-survival signaling. Cannabinoids inhibit three canonical survival cascades: (i) the PI3K (phosphoinositide 3-kinase)/Akt pathway, (ii) the Ras/Raf/MEK (mitogen-activated protein kinase kinase)/ERK (extracellular signal-regulated kinase) pathway, and (iii) the NF-κB (nuclear factor kappa-light-chain-enhancer of activated B cells) pathway (via stabilization of its inhibitor IκBα). This suppression reduces transcription of downstream survival genes including BCL-2, XIAP (X-linked inhibitor of apoptosis protein), and cIAPs (cellular inhibitor of apoptosis proteins). The convergence of pathways A–D lowers the apoptotic threshold, amplifies apoptotic execution, and overcomes chemoresistance.

### 2.5 Cannabinoid modulates tumor survival through intrinsic and microenvironmental pathways

#### 2.5.1 Cannabinoids suppress intrinsic pro-survival signaling

Malignant hematologic cells commonly sustain proliferation and evade apoptosis by persistently activating intracellular pro-survival signaling pathways. The most prominent among them are PI3K/Akt, MAPK/ERK, and NF-κB, which coordinate transcriptional and post-translational programs that enhance anti-apoptotic BCL-2 family expression, stabilize mitochondrial integrity, and dampen apoptotic signaling (Choudhary et al., 2015). In multiple myeloma, NF-κB also serves as a central node connecting tumor-intrinsic survival mechanisms with microenvironment-derived support, including inflammatory cytokine production and adhesion-mediated signaling (Hideshima et al., 2002), thereby contributing to resistance to both chemotherapeutic and targeted agents.

Available evidence indicates that cannabinoids interfere with these survival circuits through coordinated modulation of multiple signaling nodes rather than a single receptor-restricted mechanism. In Jurkat acute T-cell leukemia cells, THC induced dose-dependent apoptosis, reversible by selective CB1 and CB2 antagonists, indicating receptor-dependent signaling (Kuzma-Hunt et al., 2024). Mechanistically, THC suppressed the Raf-1/MEK/ERK/RSK pathway, reduced phosphorylation of downstream survival kinases, and promoted dephosphorylation and mitochondrial translocation of the pro-apoptotic protein Bad, thereby triggering mitochondrial apoptosis (Jia et al., 2006). Notably, this response appeared largely independent of PI3K/Akt activation, indicating that disruption of the Raf-1/MEK/ERK/RSK–Bad axis is a major mediator of THC-induced cell death in this setting.

In multiple myeloma, a similar pattern can be observed. Research shows that cannabinoids may reduce the production of anti-apoptotic proteins, such as Bcl-xL and Mcl-1, while increasing levels of pro-apoptotic proteins, such as Bax and Bak (Kuzma-Hunt et al., 2024). These changes are accompanied by adjustments in the Akt/PKB signaling pathway and an accumulation of ceramides. As a result, mitochondrial homeostasis is disrupted, making cells more susceptible to death. Cannabinoids, therefore, target not just one pathway but several survival mechanisms, including anti-apoptotic proteins, lipid-induced stress signals, and enzyme activity. This multi-pronged approach is particularly effective against drug-resistant tumor cells that rely on multiple survival mechanisms.

In addition to the old CB1/CB2 pathway, cannabinoids may also combat tumor cells through other mechanisms. In multiple myeloma, overexpression of transient receptor potential vanilloid 2 (TRPV2) makes cells more sensitive to CBD. Even more intriguing is that in resistant cells, when TRPV2 is expressed elsewhere, it actually makes the cells more receptive to treatment (Pili et al., 2014). This indicates that calcium ion influx and subsequent stress responses triggered by TRPV2 may broaden the scope of how phytocannabinoids may fight cancer.

Current research data indicate that cannabinoids may contribute to jointly inhibit pathways that rely on kinases for survival, regulate mitochondrial death factors, and activate stress-signaling pathways. In this way, cannabinoids can weaken the natural signals that keep cells alive within blood tumors. They can also suppress signaling networks that sustain drug-resistant tumor cells, thereby making traditional treatments more effective.

#### 2.5.2 Cannabinoids modulate microenvironmental protection

The tumor microenvironment is a dynamic niche composed of stromal elements, immune populations, extracellular matrix components, and metabolic conditions that together regulate tumor cell survival and therapeutic response (Maletin, 2026). In hematologic malignancies, the bone marrow and lymphoid microenvironments provide particularly strong protective support through immune suppression, cytokine signaling, metabolic adaptation, and direct cell–cell interactions (Urs et al., 2024). Preclinical evidence indicates that cannabinoids may modulate several of these microenvironmental processes through both receptor-dependent and receptor-independent mechanisms, thereby potentially weakening extrinsic survival support for malignant cells.

Within the immune compartment, cannabinoid signaling has been shown to influence both immune cell activation and immunosuppressive populations. In activated leukemic T cells, the endocannabinoid 2-AG suppressed IL-2 expression through CB1-dependent signaling, indicating that endocannabinoid pathways can directly regulate T-cell activation programs (Rockwell et al., 2006). While direct studies on leukemic T cells are limited, emerging research indicates that endocannabinoid signaling plays a complex role in hematologic malignancies (Merrien et al., 2023). Additional studies suggest that CB2-selective activation can modulate myeloid-derived suppressor cells, which are key mediators of tumor-associated immune suppression. These findings indicate that CB2-mediated signaling may partially restore antitumor immunity by reshaping immunosuppressive cell populations.

Cannabinoids have been reported to directly target tumor cells, thereby altering the immune environment. In multiple myeloma, CB2 receptors are particularly abundant in cell lines and in primary CD138+ plasma cells (Garofano and Schmidt-Wolf, 2020). Studies have shown that modulating the CB2 signaling pathway with drugs can promote CHOP expression, increase death receptor 5 (DR5) expression, activate caspase-dependent apoptosis, and simultaneously inhibit mitotic regulators such as cell-division cycle 25C (Cdc25C) and Aurora A (Feng et al., 2015). This response also disrupts the cytoskeleton, halting cell proliferation. By reducing tumor cell viability and altering tumor-derived signaling, such effects may secondarily weaken the establishment of an immunosuppressive microenvironment.

Intercellular communication within the microenvironment also contributes substantially to the dissemination of drug-resistant phenotypes. Exosomes and chemokine-mediated signaling networks allow malignant cells to exchange regulatory factors that reinforce survival programs across tumor populations. In chronic myeloid leukemia resistance models, exosomes play a key role in mediating the propagation of drug resistance through the horizontal transfer of microRNAs. For instance, a study demonstrated that exosomes from imatinib-resistant K562IR cells transfer miR-365 to sensitive K562 cells, which suppresses pro-apoptotic proteins BAX and cleaved caspase-3, thereby attenuating apoptosis and enabling chemoresistance (Ansarian et al., 2025). Separately, CBD has been shown to synergize with imatinib in reducing CML cell viability via TRPV2 channel activation, even in resistant cell lines (Maggi et al., 2022). Similar mechanisms have been implicated in AML, where exosomal miRNAs such as miR-21-5p and miR-92a contribute to chemoresistance by suppressing tumor-suppressor pathways and activating pro-survival signaling (Vandewalle et al., 2021). These observations highlight exosome-mediated communication as a potentially relevant target of cannabinoid-based sensitization strategies, although direct evidence linking cannabinoid treatment to reversal of exosome-mediated resistance remains limited.

Chemotactic pathways provide another important layer of protection for the microenvironment. The C–X–C motif chemokine ligand 12/C–X–C chemokine receptor type 4 (CXCL12/CXCR4) axis is a key regulator of tumor-cell homing to protective stromal niches (Domanska et al., 2013). In mantle cell lymphoma and chronic lymphocytic leukemia cells, the endocannabinoid 2-AG inhibited CXCL12-induced ERK1/2 phosphorylation and chemotactic migration through a CB1-dependent mechanism (Merrien et al., 2023). Because CXCR4-directed homing facilitates access to stromal survival signals, interference with this pathway may reduce niche-dependent resistance.

Metabolic crosstalk between tumor cells and the surrounding microenvironment likewise contributes to therapeutic resistance, and phytocannabinoids appear capable of disrupting this support. In glucocorticoid-resistant T-ALL, CBD suppressed mitochondrial oxidative phosphorylation and synergized with dexamethasone to inhibit both glycolysis and mitochondrial respiration; when combined with inhibition of fatty acid oxidation, this metabolic stress further depleted resistant leukemia cell populations (Olivas-Aguirre et al., 2023). In NHL, CBD inhibited mitochondrial complex I activity, depleted NADH pools, and enhanced chemotherapy-induced oxidative stress (Mould et al., 2021). Additional evidence indicates that cannabinoid signaling can also affect key metabolic regulators such as AMPK and mTOR, thereby further altering mitochondrial function and stress responses.

Overall, current evidence indicates that cannabinoids may influence the tumor microenvironment through multiple interconnected mechanisms, including modulation of immune suppression, disruption of exosome-mediated communication, inhibition of chemotactic signaling, and interference with metabolic adaptation. By weakening both tumor-intrinsic survival pathways and microenvironment-derived protective cues, cannabinoids may destabilize drug-resistant tumor ecosystems and enhance the efficacy of chemotherapy and targeted therapy in hematologic malignancies. A schematic diagram summarizing the relevant mechanisms is shown in Figure 3.

**FIGURE 3:**
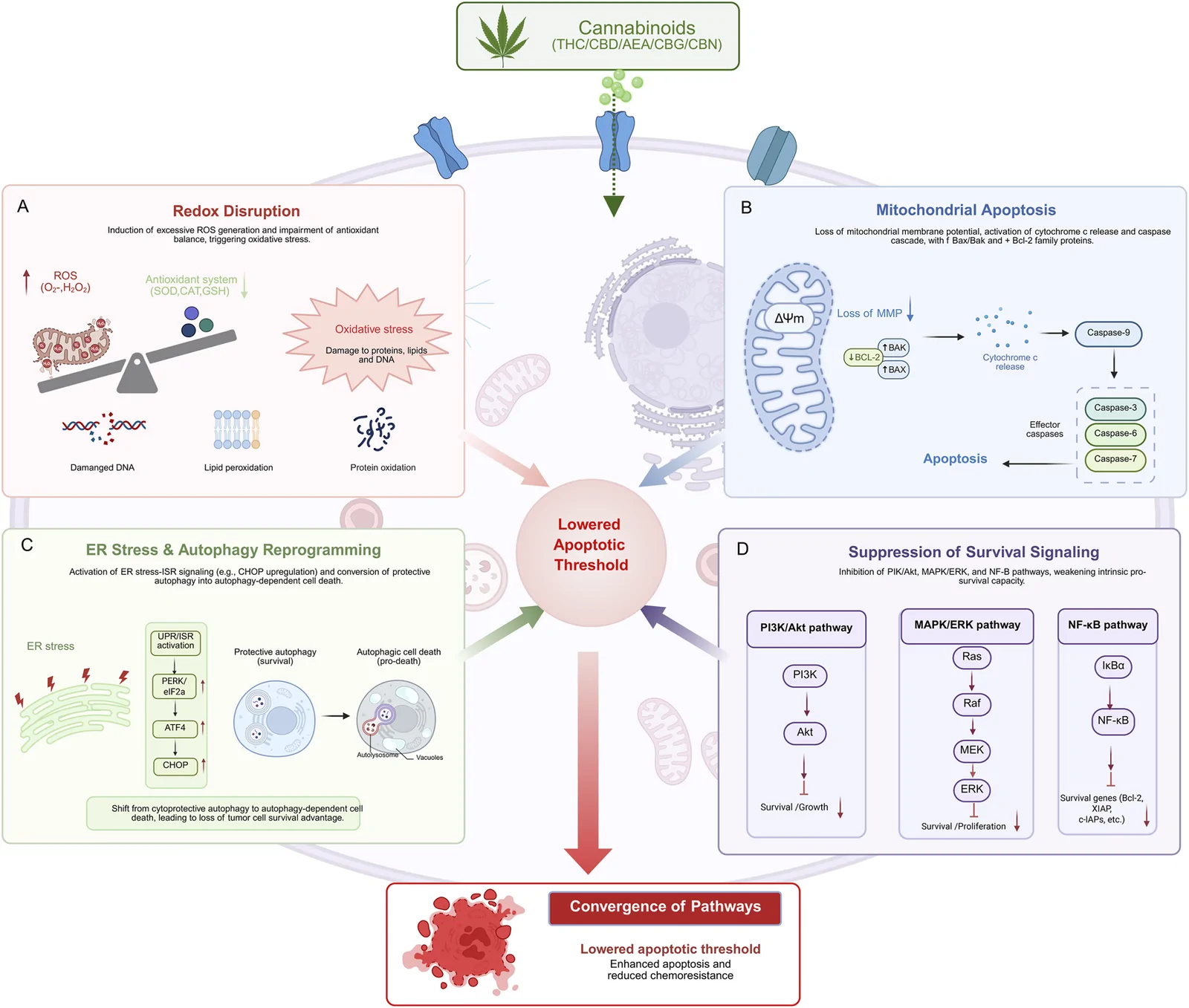
Cannabinoids disrupt tumor microenvironment-mediated mechanisms of drug resistance. Arrows indicate directional signaling or cellular trafficking, crossed-out or faded symbols represent cannabinoid-mediated inhibition or depletion, and shaded boxes denote protective anatomical niches. **(A)** Immune modulation. Cannabinoids deplete immunosuppressive cell populations—including MDSCs (myeloid-derived suppressor cells), Tregs (regulatory T cells), and M2-polarized TAMs (tumor-associated macrophages)—while simultaneously bolstering the cytotoxic activity of CD8^+^ T cells and NK (natural killer) cells, thereby restoring antitumor immune surveillance and enhancing drug sensitivity. **(B)** Inhibition of intercellular communication via extracellular vesicles. Cannabinoids suppress the release and uptake of extracellular vesicles from donor tumor cells to recipient tumor cells, thereby restricting the horizontal transfer of pro-resistance cargoes such as oncogenic miRNAs (microRNAs), DNA fragments, proteins, and other regulatory factors **(C)** Suppression of chemotaxis and niche interaction. Cannabinoids inhibit the CXCL12 (C–X–C motif chemokine ligand 12)/CXCR4 (C–X–C chemokine receptor type 4) signaling axis and its downstream MEK1/2 (mitogen-activated protein kinase kinase 1/2) → ERK1/2 cascade, thereby reducing tumor-cell homing to and retention within protective stromal niches (e.g., bone marrow) that confer cell-adhesion-mediated drug resistance. **(D)** Metabolic reprogramming. Cannabinoids disrupt oxidative phosphorylation (OXPHOS), glycolysis, and fatty acid oxidation (FAO)—thereby exacerbating intracellular oxidative stress and diminishing the metabolic adaptive capacity of tumor cells. Together, these targeted disruptions dismantle microenvironment-derived survival support, attenuate drug resistance, and broadly enhance therapeutic sensitivity and improve clinical outcomes.

## 3 Combination effects of cannabinoids and antineoplastic agents

Drug resistance in the treatment of hematologic malignancies remains a major challenge that limits clinical efficacy and long-term patient survival. As the molecular mechanisms underlying drug resistance have been increasingly elucidated, cannabinoids have emerged as a potential therapeutic strategy to overcome drug resistance in hematologic malignancies because of their ability to modulate multiple biological pathways, including ABC transporter-mediated drug efflux, endoplasmic reticulum stress, mitochondrial dysfunction, and apoptosis. Beyond their direct antitumor activity as monotherapy, cannabinoids have also shown potential to enhance antitumor efficacy, restore drug sensitivity in resistant cells, and reduce the effective doses required for treatment when used in combination with chemotherapeutic agents, targeted therapies, immunotherapies, and radiotherapy. This section will, therefore, focus on the antitumor effects and limitations of cannabinoids as monotherapy in hematologic malignancies, while emphasizing recent research progress on their use in combination with existing antineoplastic therapies, particularly their synergistic roles in enhancing therapeutic efficacy and overcoming drug resistance.

### 3.1 Anti-tumor biological effects of cannabinoids as monotherapy

Existing studies indicate that cannabinoids, when used as monotherapy, exhibit anti-tumor activity in various hematologic tumor models; however, the magnitude of their effects is highly compound-specific and cell type-dependent. Gallily et al. reported that CBD induces dose-dependent apoptosis in HL-60 cells, a model of AML (Gallily et al., 2003). Powles et al. further found that high concentrations of THC (60 μM) induced pronounced G_1_/S-phase cell-cycle arrest in CEM, HEL-92, and HL-60 cells (Powles et al., 2005). More recently, Medrano et al. confirmed that the synthetic cannabinoid derivative WIN-55,212-2 exerts concentration-dependent cytotoxic effects in AML cell lines, including HL-60, MV-4-11, KG-1a, and U937, and in primary leukemic blast cells derived from patients with AML (Medrano et al., 2024).

Some studies have also demonstrated antileukemic effects of cannabinoids as monotherapy in preclinical models. Olivas-Aguirre et al. observed in Jurkat and CCRF-CEM cells with ALL that the anti-tumor effects of CBD are closely associated with mitochondrial dysfunction (Olivas-Aguirre et al., 2019). Moreover, it does not cause any toxic side effects on normal immune cells. Thamm et al. added that both CBD and THC can inhibit the viability of various blood tumor cells by disrupting mitochondrial function (Thamm et al., 2026).

However, the clinical application of cannabinoids as monotherapy still faces substantial limitations. First, currently, there is a lack of high-quality clinical trial evidence supporting their efficacy as monotherapy in patients with hematologic malignancies, and the therapeutic exposure range and the magnitude of efficacy relative to standard treatments remain incompletely defined. Second, the immunomodulatory effects of cannabinoids are highly context-dependent. It reported that at non-toxic concentrations (≤8 μM), CBD had no significant adverse effects on the cytotoxic activity or cytokine secretion of CD19-CAR T cells (Chantarat et al., 2024). However, it did inhibit their proliferative capacity, indicating a potential impact on the persistence of anti-tumor effects. Nagarkatti et al. noted that THC tends to suppress cellular immunity and induce immune tolerance; in contrast, (Hegde et al., 2008) Rieder et al. reported that THC can enhance cellular immunity. Collectively (Rieder et al., 2010), these findings indicate that the net effect of cannabinoid monotherapy in complex tumor microenvironments may be influenced by the drug type, dose, immune status, and tumor background.

To overcome the limitations of monotherapy, researchers have begun exploring combination strategies involving different cannabinoids. Nabissi et al. found that in U266 and RPMI8226 multiple myeloma cells, the combination of THC (12.5 μM) and CBD (12.5 μM) produced stronger cytotoxic effects than either agent alone, and it significantly inhibited tumor cell migration (Nabissi et al., 2016). Besser et al. further demonstrated, in a *NOTCH1*-mutant T-ALL model, that single cannabinoids or dual-drug combinations exhibited only limited cytotoxicity. In contrast, a triple combination of 331-18A, CBDV, and CBD induced apoptosis in up to 88.29% ± 4.31% of cells and significantly inhibited tumor growth while prolonging survival *in vivo* (Besser et al., 2024). Kadriya et al. reported that in THP-1 acute monocytic leukemia cells, both CBN and CBG inhibited cell growth and induced apoptosis (Kadriya et al., 2024); however, their effective concentration ranges differed markedly, indicating that the efficacy of cannabinoid combinations may depend on rational ratio design and dose optimization.

In summary, although cannabinoids as monotherapy have demonstrated antitumor activity in various hematologic malignancy models, their therapeutic potency remains limited, and clinical validation is insufficient. Compared with monotherapy, combination strategies involving cannabinoids and existing antineoplastic therapies appear more promising for enhancing efficacy and overcoming resistance. A summary of the relevant content is shown in Table 1.

**TABLE 1:** Antitumor effects of cannabinoids as monotherapy.

Cannabinoid investigated	Hematologic malignancy	Experimental model	Principal findings	Level of evidence	References
Cannabinoid derivative WIN-55,212-2	Acute myeloid leukemia (AML)	Primary blasts from patients with AML	Produces concentration-dependent cytotoxicity	*Ex vivo*	Medrano et al., 2024
Cannabinol (CBN)	Acute monocytic leukemia	THP-1 cells	Produces dose- and time-dependent effects; more sensitive in inducing apoptosis	*In vitro*	Kadriya et al., 2024
Cannabigerol (CBG)	Acute monocytic leukemia	THP-1 cells	Produces dose- and time-dependent effects; more effective in inhibiting cellular metabolic activity	*In vitro*	Kadriya et al., 2024
Cannabinoid derivative WIN-55,212-2	AML	NSG mouse HL-60 xenograft model	Significantly reduces leukemia cell infiltration in bone marrow, peripheral blood, and spleen; improves splenomegaly; no significant toxicity to normal immune cells	Animal	Medrano et al., 2024
331-18A+ cannabidivarin (CBDV) + CBD	NOTCH1-mutant acute T-lymphoblastic leukemia	NOTCH1-mutant acute T-lymphoblastic leukemia mouse model	Single agent or dual combination produces only limited cytotoxicity; triple combination induces up to 88.29% apoptosis, significantly inhibits tumors *in vivo*, and prolongs survival	Animal	Besser et al., 2024
Cannabidiol (CBD)	AML	HL-60 cells	Induces dose-dependent apoptosis	*In vitro*	Gallily et al., 2003
Tetrahydrocannabinol (THC)	Acute T-lymphoblastic leukemia, AML	CEM cells, HEL-92 cells, and HL-60 cells	High concentration (60 μM) induces significant G1/S cell-cycle arrest	*In vitro*	Powles et al., 2005
CBD	Acute lymphoblastic leukemia (ALL)	Jurkat cells and CCRF-CEM cells	Exhibits antitumor activity, possibly associated with mitochondrial dysfunction	*In vitro*	Olivas-Aguirre et al., 2019
THC + CBD	Multiple hematological malignancies	Multiple hematological tumor cell lines	Inhibits tumor cell activity by inducing mitochondrial dysfunction	*In vitro*	Thamm et al., 2026
THC + CBD	Multiple myeloma	U266, RPMI8226 cells	Combination of 12.5 μM THC and 12.5 μM CBD yields far stronger cytotoxicity than single agents, and significantly inhibits tumor cell migration	*In vitro*	Nabissi et al., 2016

### 3.2 Combination effects of cannabinoids with different types of antineoplastic drugs

#### 3.2.1 Combination effects of cannabinoids and chemotherapy drugs

Chemotherapy represents a cornerstone of treatment for hematologic malignancies; however, primary or acquired drug resistance, along with dose-limiting toxicity, substantially compromise clinical outcomes. A growing body of *in vitro* evidence indicates that combining cannabinoids with commonly used chemotherapeutic agents can enhance antitumor effects and increase the sensitivity of drug-resistant tumor cells to chemotherapy. In studies that quantitatively evaluated drug interactions, the Chou–Talalay combination index (CI) method was used, with CI < 1 indicating synergism, CI = 1 an additive effect, and CI > 1 antagonism. It should be noted that these findings have been generated almost exclusively in cell line models and have not been validated in *in vivo* models of drug-resistant hematologic malignancies.

The combination of CBD and dexamethasone has been shown to enhance the response of drug-resistant cells to hormonal therapy. In an ALL model, Olivas-Aguirre et al. utilized dexamethasone-sensitive CCRF-CEM cells and dexamethasone-resistant Jurkat cells to compare the cytological effects of CBD, dexamethasone, and their combination. Combination treatment produced greater inhibition of proliferation and induction of apoptosis in both cell types relative to monotherapy; notably, this potentiating effect was more pronounced in dexamethasone-resistant Jurkat cells, indicating that CBD can amplify the cytotoxic effects of hormonal therapy in a drug-resistant context (Olivas-Aguirre et al., 2024). These findings indicate that CBD may enhance the effects of glucocorticoid therapy in resistant leukemia cells. Beyond glucocorticoids, the combination of cannabinoids with vincristine has also been shown to improve the chemotherapeutic response of leukemia cells. Scott et al. evaluated the antitumor effects of cannabinoid–vincristine combinations using CEM and HL60 cell lines as models. CBD + THC exhibited strong synergistic interactions with vincristine, with CI as low as 0.29 in HL60 cells. Even at markedly reduced doses, the combination achieved cytotoxic effects comparable to those of high-dose vincristine monotherapy, thereby circumventing the dose-dependent neurotoxicity that limits vincristine’s clinical utility. Further analysis revealed that sub-toxic concentrations of CBD + THC reduced the IC_50_ of vincristine by 12.5-fold, significantly enhancing chemosensitivity (Scott et al., 2017). Importantly, the study identified the sequence of administration as a critical determinant of synergistic efficacy: pre-treatment with vincristine to initiate tumor cell damage stress, followed by cannabinoids to suppress damage repair and pro-survival signaling, significantly enhanced apoptosis induction and reduced the survival of resistant clones; the reverse sequence diminished efficacy and may even promote resistance. These findings indicate that cannabinoid–vincristine combinations can substantially enhance chemotherapeutic cytotoxicity, and that optimizing the administration sequence can further amplify the synergistic effects, offering a potential strategy for overcoming chemotherapy resistance in relapsed and refractory hematologic malignancies.

The combination of cannabinoids with cytarabine also shows potential for enhancing the chemotherapeutic response. An *in vitro* study by Liu et al. demonstrated that the combination of THC and cytarabine significantly enhanced leukemia cells’ response to conventional chemotherapy. In three leukemia cell lines—CEM, HL60, and MOLT4—the Chou–Talalay CI for the THC–cytarabine regimen was less than 1 in all cases, indicating synergistic cytotoxicity. Pre-treatment with low, non-cytotoxic concentrations of THC significantly reduced the IC_50_ of cytarabine, thereby sensitizing leukemia cells to chemotherapy (Liu et al., 2008). Consistent with these findings, Scott et al. reported that sub-toxic concentrations of cannabinoids combined with cytarabine similarly reduced the IC_50_ of the chemotherapeutic agent and produced synergistic cytotoxic effects, leading to marked chemosensitization of leukemic cells and potentially mitigating the risks of insufficient efficacy and resistance associated with dose limitations (Scott et al., 2017). For patients with relapsed or refractory acute leukemia in whom prior drug exposure is substantial and further dose escalation is constrained, cannabinoid combination strategies may offer a means of enhancing the response to existing treatments.

In the chemotherapy of lymphoma, cannabinoids have also been shown to enhance tumor cell sensitivity to conventional chemotherapeutic agents. Saba Omer et al. utilized canine B-cell non-Hodgkin lymphoma (B-NHL) cell lines as a model. They evaluated three representative cannabinoids—the endocannabinoid AEA, the phytocannabinoid CBD, and the synthetic cannabinoid WIN-55212-2—in combination with the core components of the CHOP regimen (cyclophosphamide, doxorubicin, vincristine, and prednisolone) and lomustine. Sub-IC_50_ concentrations of all three cannabinoids produced synergistic cytotoxic effects (CI < 1) with all tested chemotherapeutic agents, with particularly pronounced synergy observed in combination with vincristine, lomustine, and prednisolone (Omer et al., 2025). Notably, the synergistic effects were strictly dose-dependent, with synergistic effects (CI < 1) observed within the sub-IC_50_ range, whereas antagonistic interactions (CI > 1) emerged at higher-dose combinations. These findings indicate that cannabinoids can produce synergistic anti-tumor effects with conventional lymphoma chemotherapy drugs by targeting core pathways of chemotherapy resistance, thereby enhancing chemosensitivity and reversing resistance phenotypes. This work also provides experimental support for optimizing chemotherapy dosing and reducing non-specific toxicity, offering a new research direction for the treatment of relapsed and refractory lymphoma.

Overall, existing evidence indicates that cannabinoid–chemotherapy combinations may enhance the response of hematologic malignancy cells to existing treatments, with particular promise in drug-resistant models and relapsed or refractory settings. Although current evidence remains largely derived from *in vitro* and preclinical animal studies, the potential of these combination strategies to address drug resistance in hematologic malignancies warrants further investigation. A summary of the relevant content is shown in Table 2.

**TABLE 2:** Combination effects of cannabinoids and chemotherapy drugs.

Cannabinoid investigated	Chemotherapeutic agent	Mechanism of action/molecular target	Hematologic malignancy	Experimental model	Principal findings	Level of evidence	References
CBD	Dexamethasone	Glucocorticoid receptor (GR/NR3C1) agonist; nuclear-receptor-mediated gene regulation	ALL	Primary samples from patients with ALL (newly diagnosed and relapsed/refractory)	Combination shows superior proliferation inhibition and apoptosis induction over single agents; partially overcomes glucocorticoid resistance; minimal effects on normal hematopoietic cells	*Ex vivo*	Olivas-Aguirre et al., 2019
CBD	Dexamethasone	Glucocorticoid receptor (GR/NR3C1) agonist	ALL	CCRF-CEM cells and Jurkat cells (drug-resistant)	Combination shows superior proliferation inhibition and apoptosis induction over single agents; synergy is more pronounced in drug-resistant Jurkat cells	*In vitro*	Olivas-Aguirre et al., 2023
CBD	Vincristine	Binds β-tubulin and inhibits microtubule polymerization, thereby leading to mitotic arrest	ALL, promyelocytic leukemia	CEM cells and HL60 cells	Strong synergy (CI = 0.29 in HL60); sub-toxic cannabinoid concentrations reduce vincristine IC_50_ by 12.5-fold; sequence of administration is critical: Chemotherapy followed by cannabinoid enhances efficacy, while reverse order attenuates effect	*In vitro*	Scott et al., 2017
THC	Cytarabine	Nucleoside analog; inhibits DNA polymerase; incorporated into DNA thereby resulting blocks DNA synthesis	All, AML	CEM cells, HL60 cells, and MOLT4 cells	Pre-treatment with non-cytotoxic low-concentration THC significantly reduces cytarabine IC_50_, producing synergistic cytotoxicity (CI < 1)	*In vitro*	Scott et al., 2017
AEA/CBD/WIN-55212–2	CHOP regimen (cyclophosphamide, doxorubicin, vincristine, prednisolone) + lomustine	DNA alkylation/crosslinking, topoisomerase II inhibition combined with DNA intercalation, tubulin-microtubule inhibition, and glucocorticoid receptor agonism	B-cell non-Hodgkin lymphoma (B-NHL)	Canine B-cell lymphoma cell lines	Sub-IC_50_ concentrations of cannabinoids produce synergistic cytotoxicity with all tested chemotherapeutic agents (CI < 1); synergy is strictly dose-dependent, with antagonism observed at high-dose combinations	*In vitro*	Omer et al., 2025

#### 3.2.2 Combination effects of cannabinoids and targeted therapies

Targeted therapy has now become an important component of precision medicine for blood cancers. However, once resistance emerges, bypass pathways are activated, and the tumor continues to evolve. These factors mean that the benefits of targeted therapy are not long-lasting. Recent research has found that substances such as cannabinoids, particularly CBD, can work in conjunction with various forms of targeted therapy. By regulating oxidative stress, mitochondrial function, protein balance, and cell survival signals, they can enhance treatment effectiveness and make resistant cells sensitive to drugs again.

The enhanced antitumor effects of cannabinoids combined with proteasome-targeted therapies have been consistently demonstrated in multiple myeloma models. Morelli et al. showed that CBD and bortezomib act more potently together than individually to suppress myeloma cell proliferation and induce cell death, with the CBD–bortezomib combination significantly enhancing cytotoxicity in patient-derived CD138+ primary plasma cells while exerting relatively limited effects on normal CD34^+^ progenitor cells, indicating potential therapeutic selectivity (Morelli et al., 2014). Extending these findings, Nabissi et al. evaluated combinations of cannabinoids with the immunoproteasome inhibitor carfilzomib in U266 and RPMI8226 cell lines (Nabissi et al., 2016). They demonstrated that CBD and THC markedly enhanced carfilzomib-mediated cell killing and effectively reversed interferon-γ (IFN-γ)-driven resistance by suppressing the immunoproteasome β5i subunit. This cannabinoid–carfilzomib regimen also significantly inhibited myeloma cell migratory capacity by downregulating the C–X–C chemokine receptor type 4 (CXCR4) and CD147. Mechanistically, the cannabinoid combination reduced β5i expression at both the transcriptional and translational levels while complementing carfilzomib-induced caspase-3-dependent apoptosis, jointly amplifying antitumor activity (Ramer and Hinz, 2025). At the same time, it increases caspase-3-dependent cell death induced by carfilzomib. This, in turn, improves the anti-tumor effect. These studies indicate that cannabinoids may enhance the efficacy of proteasome inhibitors by regulating proteasome-related pathways. These preclinical findings raise the possibility that this approach could, if successfully translated, help overcome certain resistance mechanisms *in vitro* and might limit tumor cell migration in experimental models.

Beyond multiple myeloma, the combination of cannabinoids with TKIs has also shown therapeutic promise. Maggi et al. reported that CBD can enhance the sensitivity of CML cells to imatinib (Maggi et al., 2022). Donchev et al. further demonstrated, in the imatinib-resistant K-562R cell line, that the combination of CBD and imatinib significantly restored cytotoxicity, with the Chou–Talalay CI decreasing to 0.69. The dose-reduction index (DRI) for imatinib reached 9.18, indicating that this strategy may improve the response of resistant cells while reducing the overall drug exposure (Donchev et al., 2026). Although these studies remain at the preclinical stage, they provide preliminary evidence supporting the combination of cannabinoids with targeted therapy in the setting of CML resistance. Cannabinoids have also been investigated in combination with inhibitors of the B-cell receptor signaling pathway to enhance therapeutic efficacy in NHL. Strong et al. found in an NHL model that the combination of CBD with the Bruton’s tyrosine kinase (BTK) inhibitors ibrutinib or zanubrutinib significantly reduced cell viability, with greater effects than either agent alone (Strong et al., 2018). These findings indicate that cannabinoids may enhance the sensitivity of B-cell-derived tumors to BTK inhibitors by acting on cellular stress and survival pathways that intersect with BTK signaling, thereby offering a potential combination strategy for relapsed or refractory B-NHL.

In summary, the combination of cannabinoids with targeted therapies has demonstrated promising preclinical sensitization effects across multiple subtypes of hematologic malignancies; however, the underlying mechanisms of action, applicable disease subtypes, and optimal combination regimens remain to be fully elucidated. Future studies should further evaluate the translational value of this approach by comprehensively considering the molecular subtypes, resistance mechanisms, and pharmacokinetic characteristics. A summary of the relevant content is shown in Table 3.

**TABLE 3:** Combination effects of cannabinoids and targeted therapies.

Cannabinoid investigated	Targeted agent	Mechanism of action/molecular target	Malignancy	Experimental model	Principal findings	Level of evidence	References
CBD/THC	Bortezomib/Carfilzomib	Proteasome inhibitor (20S proteasome; β5 subunit; carfilzomib also inhibits immunoproteasome β5i)	Multiple myeloma	Primary CD138+ plasma cells from patients	Synergistic pro-apoptotic effect; minimal damage to normal CD34^+^ progenitors; reverses IFN-γ-mediated resistance	*Ex vitro*	Morelli et al., 2014; Nabissi et al., 2016
CBD/THC	Bortezomib/Carfilzomib	Proteasome inhibitor (20S proteasome; β5 subunit; carfilzomib also inhibits immunoproteasome β5i)	Multiple myeloma	Multiple myeloma cell lines	Synergistic pro-apoptotic effect; reverses IFN-γ-mediated resistance; inhibits tumor cell migration by downregulating CXCR4 a nd CD147	*In vitro*	Morelli et al., 2014; Nabissi et al., 2016
CBD	Imatinib	BCR-ABL1 tyrosine kinase inhibitor (also inhibits c-KIT, PDGFR)	Chronic myeloid leukemia	K-562R imatinib-resistant cells	Significantly restores sensitivity in resistant cells (CI = 0.69); dose reduction index reaches 9.18, achieving enhanced efficacy and reduced toxicity	*In vitro*	Donchev et al., 2026
CBD	Ibrutinib/Zanubrutinib	Bruton’s tyrosine kinase (BTK) inhibitor	B-NHL	B-NHL cell lines	Synergistic inhibition of tumor cell activity, far superior to single-agent treatment	*In vitro*	Strong et al., 2018

#### 3.2.3 Combination effects of cannabinoids and immunotherapeutic agents

Immunotherapy has demonstrated considerable efficacy in the treatment of various hematologic malignancies and certain solid tumors. However, its clinical efficacy is still limited by heterogeneous immune checkpoint expression, an immunosuppressive tumor microenvironment, immune escape, and treatment-related adverse events. Compared with chemotherapy and targeted therapy, direct evidence supporting the combination of cannabinoids with immunotherapeutic agents in hematologic malignancies remains limited. Therefore, the current significance of this strategy lies mainly in its potential to complement existing immunotherapeutic regimens by improving treatment tolerability or modulating immune-related pathways.

The combination of cannabinoids with immune checkpoint inhibitors has gained increasing attention. Kim et al. reported that CBD enhanced the efficacy of programmed cell death protein 1/programmed death-ligand 1 (PD-1/PD-L1) blockade, including atezolizumab, in a triple-negative breast cancer model. This effect was associated with activation of the cyclic GMP-AMP synthase-stimulator of interferon genes (cGAS-STING) pathway and increased PD-L1 expression in tumor cells. Although this evidence was derived from a solid tumor model, it provides a rationale for exploring whether cannabinoids may improve responsiveness to immune checkpoint inhibition in hematologic malignancies (Kim et al., 2024). Given the involvement of the cGAS-STING pathway in the immune regulation of hematologic tumors such as T-cell large granular lymphocytic leukemia and NK/T-cell lymphoma, this combination strategy may have potential translational relevance, but further disease-specific validation is required.

Cannabinoids may also contribute to combination therapy by improving treatment tolerability. Gavriatopoulou et al. reported an integrated safety analysis of 437 patients with multiple myeloma treated with selinexor in clinical trials. Cannabinoids, including cannabis sativa and dronabinol, were used as supportive care in 6% of patients to manage decreased appetite and associated weight loss. Cannabinoids were also reported to be effective in mitigating nausea and vomiting, with a 77% AE resolution rate when used within 5 days of onset (Gavriatopoulou et al., 2020). Although these findings do not directly demonstrate enhanced antitumor immunity, they indicate that cannabinoids may indirectly support immunotherapy-containing regimens by improving tolerability, maintaining treatment intensity, and promoting patient adherence. This supportive effect is particularly important in multiple myeloma, as this disease often requires long-term maintenance therapy.

Nevertheless, the use of cannabinoids in combination with immunotherapy should be approached with caution, as some cannabinoids, particularly THC, may exert immunosuppressive effects. Rieder et al. showed that THC induced an immunosuppressive state in a 4T1 murine breast cancer model, thereby promoting tumor growth and metastasis (Rieder et al., 2010). Further preclinical studies have shown that THC-treated antigen-presenting cells exhibited a reduced capacity to activate T cells (Henriquez et al., 2017). In a clinical setting, Taha et al. reported that concurrent cannabis use was associated with reduced efficacy in patients receiving anti-PD-1 therapy with nivolumab (Taha et al., 2019). The distinction between CBD and THC is, therefore, particularly important. Yekhtin et al. reported that CBD and THC exert different effects on macrophage phenotypes, with THC upregulating the macrophage surface marker F4/80 and CBD significantly downregulating its expression (Yekhtin et al., 2022). These divergent effects indicate that cannabinoid-based combination strategies should be evaluated according to the cannabinoid subtype, dose, timing of administration, and tumor immune background.

Overall, combining cannabinoids with immunotherapy drugs for treating blood cancer could be promising. However, the effectiveness of this approach needs to be clearly demonstrated. We need to conduct more clinical trials across different disease types to determine whether this method is feasible and to ensure safety. If it proves effective, it would be a real benefit for patients. A summary of the relevant content is shown in Table 4.

**TABLE 4:** Combination effects of cannabinoids and immunotherapeutic agents.

Cannabinoid investigated	Immunotherapeutic agent	Mechanism of action/molecular target	Experimental model	Principal findings	Level of evidence	References
CBD	Atezolizumab	Anti-PD-L1 (CD274) monoclonal antibody	Breast cancer model	Enhanced anti-PD-L1 efficacy via cGAS-STING activation; increased PD-L1 expression	*In vitro*	Kim et al., 2024
Cannabis (unspecified)	Nivolumab	Anti-PD-1 (PDCD1) monoclonal antibody	Retrospective clinical cohort	Concurrent cannabis use associated with reduced nivolumab efficacy	Clinical	Taha et al., 2019
Cannabis sativa, dronabinol	Selinexor	XPO1 (exportin-1/CRM1) inhibitor	437 patients (integrated safety analysis)	Supportive care; 77% AE resolution for nausea/vomiting; improved tolerability	Clinical	Gavriatopoulou et al., 2020

#### 3.2.4 Combination effects of cannabinoids and radiation therapy

Radiation therapy is an effective modality for certain hematologic malignancies, particularly for local disease control and symptom palliation. Tumors often do not respond well to this treatment, and it can also damage healthy tissues. Therefore, its use is indeed limited. In recent years, non-psychoactive cannabinoids have drawn increasing attention as potential radiosensitizers. Gallily et al. were among the first to demonstrate in human AML HL-60 cells that natural CBD and its synthetic derivative, CBD-dimethylheptyl (CBD-DMH), when combined with γ-irradiation, significantly enhanced cytotoxicity while exerting minimal impact on normal hematopoietic cells, indicating a degree of tumor selectivity (Gallily et al., 2003). Although these findings are primarily derived from *in vitro* studies, they provide preliminary evidence supporting a radiosensitizing role for cannabinoids in hematologic malignancies.

Evidence from solid tumor models has further provided indirect support for this approach. Ivanov et al. (2017) demonstrated in human glioblastoma cells that the combination of CBD and γ-irradiation significantly enhanced anti-tumor effects while causing minimal damage to normal neural stem cells and astrocytes (Ivanov et al., 2017). Scott et al. reported that pre-treatment with a combination of THC and CBD before radiotherapy increased tumor cell sensitivity to ionizing radiation (Scott et al., 2014). Consistent with these findings, Scott et al. observed in a mouse orthotopic glioma model that the combination of THC and CBD with radiotherapy yielded superior outcomes compared with radiotherapy alone (Scott et al., 2014). Although these studies were not specifically conducted in hematologic malignancy models, they collectively indicate that cannabinoids may enhance the response to radiotherapy by augmenting oxidative stress, impairing DNA repair, or modulating cell-death pathways.

Although current evidence on the use of cannabinoids to enhance radiotherapeutic efficacy in hematologic malignancies remains limited, the existing findings provide a foundation for further validation in relevant models such as leukemia and lymphoma. Future studies should focus on optimizing combination regimens and treatment strategies and quantitatively defining potential synergistic interactions, thereby advancing their translational and clinical application.

## 4 Challenges and outlook

Growing preclinical evidence indicates that cannabinoids can modulate multiple processes involved in therapeutic resistance in hematologic malignancies, including drug efflux, apoptosis, oxidative stress, autophagy, pro-survival signaling, and tumor–microenvironment interactions. However, their effects vary considerably across experimental systems and are not consistently favorable. This variability may reflect differences in the cannabinoid subtype, receptor expression, tumor biology, host immune status, drug concentration, exposure duration, and experimental conditions. Most available evidence remains preclinical, while direct clinical evidence in hematologic malignancies is limited. Clinical studies in oncology have largely focused on symptom management rather than antineoplastic activity (Bar-Sela et al., 2022), and whether cannabinoids can improve treatment response or overcome therapeutic resistance in patients with hematologic malignancies remains unclear.

The inconsistent findings are particularly evident in immune-related settings. Cannabis use during immune checkpoint blockade has been associated with poorer treatment responses in some retrospective cohorts (Taha et al., 2019), while CBD has been reported to preserve CAR-T-cell cytotoxicity but reduce their proliferative capacity. Although these observations do not establish a detrimental clinical effect, they indicate that direct antitumor activity may coexist with modulation of immune effector function. Similar discrepancies have been observed in animal studies. Cannabinoids have reduced tumor burden or prolonged survival in selected leukemia models (Besser et al., 2024; Medrano et al., 2024), whereas THC has promoted tumor progression or impaired tumor-rejection responses in some immunocompetent models through CB2-associated immunosuppression and altered cytokine signaling (Rieder et al., 2010). Because many favorable studies were conducted in immunodeficient xenograft models, these systems cannot fully capture cannabinoid-mediated interactions with an intact immune system (Dobovišek et al., 2024). Moreover, differences between murine and human immune responses may further limit the translation of findings from animal models. This issue is particularly relevant to hematologic malignancies, in which therapeutic responses are closely influenced by the bone marrow and lymphoid microenvironments.

Experimental and pharmacological differences further contribute to the heterogeneity of reported outcomes. Cannabinoid responses can depend strongly on the concentration and exposure time and may display biphasic effects. Lower concentrations may exert limited cytotoxicity or transiently enhance resistance-associated pathways, whereas higher concentrations or prolonged exposure may inhibit drug transporters, disrupt mitochondrial function, and promote apoptosis (Lee and Chae, 2026). Conventional cell-line models also fail to reproduce key features of the bone marrow niche, including hypoxia, stromal protection, immune regulation, and clonal heterogeneity, and therefore, they may not fully reflect treatment responses in primary hematologic malignancies (Pressman and Hayes, 2026). Differences among cannabinoid compounds add further complexity, as THC, CBD, synthetic agonists, and non-psychoactive derivatives differ in receptor selectivity, pharmacokinetics, and immunological effects.

Pharmacokinetic limitations represent an additional challenge for clinical translation. Cannabinoids generally exhibit variable bioavailability and undergo extensive metabolism, leading to substantial variability in systemic exposure depending on the compound, formulation, dose, and route of administration. This is particularly relevant because concentrations producing chemosensitizing effects *in vitro* may not always be achievable in human circulation at clinically tolerated doses (Mseddi et al., 2026). Their lipophilic properties also allow penetration of the blood–brain barrier, although the extent of exposure varies among individual cannabinoids. Differences in the regulatory status and standardization of cannabinoid products may further complicate their clinical development. CBD and related compounds can affect CYP3A4, CYP2C19, CYP2D6, and membrane drug transporters, potentially altering the exposure or toxicity of anticancer agents (Turck et al., 2022). Although such interactions may contribute to enhanced therapeutic effects in some combinations, increased drug exposure may also exacerbate toxicity, particularly for agents with a narrow therapeutic index. These potential drug–drug interactions are particularly relevant to combination therapy and should be carefully considered when translating preclinical findings into clinical settings.

Future studies should focus on improving the clinical relevance of experimental models and defining the conditions under which cannabinoid effects are reproducible. Patient-derived cells, patient-derived organoid models, patient-derived xenografts (PDXs), bone marrow stromal co-cultures, physiologically relevant hypoxic conditions, and immunocompetent animal models should be prioritized, together with greater standardization of cannabinoid formulation, dose, exposure time, and administration route (Hermush et al., 2025). These patient-derived models may better preserve disease heterogeneity and provide a more clinically relevant platform for evaluating treatment response (Sadrykia et al., 2026). These approaches will allow the direct effects on malignant cells to be evaluated alongside microenvironmental and immune influences and will help distinguish robust pharmacological responses from effects restricted to specific experimental settings.

At the translational level, emphasis should be placed on mechanism-guided combination therapy and biomarker-guided patient selection as part of a precision medicine strategy. Integration of patient-derived models with molecular and pharmacological biomarkers may further support individualized evaluation of cannabinoid-based treatment strategies (Shittu et al., 2024). The dose, treatment sequence, and therapeutic partner should be carefully optimized, particularly in combination with immunotherapy, where antitumor activity may coexist with immune modulation (Ramer and Hinz, 2025). Biomarkers such as CB1/CB2 expression, ABC transporter profiles, and key resistance-related molecular features may help identify responsive subgroups. The development of receptor-selective CB1/CB2 agonists or antagonists, along with cannabinoid derivatives with reduced psychoactive effects, may further improve therapeutic specificity and safety (Banchhor et al., 2025). Nanotechnology-based delivery systems may also help overcome the poor aqueous solubility, variable bioavailability, and non-specific distribution of cannabinoids and facilitate tumor-targeted delivery.

Early clinical studies should prioritize pharmacokinetics, drug–drug interactions, safety, and pharmacodynamic evidence of resistance modulation before broader efficacy testing. Importantly, current clinical studies of cannabinoids in oncology remain largely focused on supportive care and symptom management, whereas direct evaluation of their antitumor or resistance-modulating effects remains limited, particularly in hematologic malignancies (Ankit Singh, 2025). Future clinical trials should, therefore, incorporate biomarker-defined patient populations and pharmacodynamic endpoints to determine whether the mechanisms identified preclinically can be translated into clinically meaningful treatment sensitization.

Overall, current evidence supports the potential of cannabinoids to modulate therapeutic resistance in hematologic malignancies, but their effects are highly dependent on compound type, exposure conditions, tumor biology, and immune context. The conflicting findings reported across studies highlight the need to define the specific settings in which these effects are reproducible and therapeutically relevant. Future work should, therefore, focus on clinically achievable exposures, rational cannabinoid–drug combinations, and validation in patient-derived and immune-competent models to better identify the disease contexts in which cannabinoid-based strategies may provide meaningful benefit.

## 5 Conclusion

In summary, the application of cannabinoids in reversing drug resistance and combination therapy for hematological malignancies remains in the early translational stage. Current evidence indicates that cannabinoids may modulate drug-resistant phenotypes through multiple mechanisms and produce synergistic effects with various antineoplastic treatment modalities, indicating their potential as an adjunctive therapeutic strategy. However, to translate this potential into tangible clinical benefits, future research should shift its focus from merely validating antineoplastic activity to comprehensively elucidating their pharmacological feasibility, safety profiles, optimal combination scenarios, and suitable patient populations. If a continuous and reliable chain of evidence is established linking mechanistic studies, pharmacological evaluations, biomarker discovery, and rigorously designed clinical trials, cannabinoid-based therapeutic strategies will be more likely to be implemented in the clinical treatment of hematologic malignancies, leading to the development of practical treatment regimens and ultimately providing new therapeutic options for patients with relapsed or refractory disease.
